# Blood–brain barrier permeability and electroconvulsive therapy: a systematic review

**DOI:** 10.1017/neu.2023.48

**Published:** 2023-10-16

**Authors:** Christoffer C. Lundsgaard, Krzysztof Gbyl, Poul Videbech

**Affiliations:** 1 Center for Neuropsychiatric Depression Research, Mental Health Centre Glostrup, Denmark; 2 Institute of Clinical Medicine, Faculty of Health and Medical Sciences, University of Copenhagen, Copenhagen, Denmark

**Keywords:** Electroconvulsive therapy, electroshock, major depressive disorder, mood disorders, blood–brain barrier

## Abstract

**Objective:**

The cause of cognitive side effects after electroconvulsive therapy (ECT) is largely unknown. Alterations in the blood–brain barrier (BBB) have been considered in several recent ECT studies. We therefore found it worthwhile to perform a systematic review of the literature to examine if electrically induced seizures affect the permeability of the BBB.

**Methods:**

PubMed/MEDLINE and Embase were searched 16 November 2022. Studies with a direct measurement of BBB permeability in animals treated with modified electroconvulsive stimulation (ECS) and in humans treated with ECT were included. Synthesis of results was narrative due to the low number of studies and differences in study designs.

**Results:**

Four animal and two human (31 participants) studies were included. In animals, two studies found increased BBB permeability to some smaller molecules after modified ECS, while the two other studies found marginally increased or unchanged permeability to albumin after treatment. In contrast, the human studies did not find increased BBB permeability to smaller molecules or albumin after ECT.

**Conclusion:**

Animal but not human studies support increased BBB permeability to some smaller molecules after electrically induced seizures. However, this conclusion is confined by the low number of studies and the lack of studies applying state-of-the-art methods. More studies using modern approaches to measuring of BBB permeability are warranted.

**Funding and Registration:**

The study was founded by *Mental Health Services in the Capital Region of Denmark* (grant number 61151-05) and was registered on PROSPERO before data extraction was initiated (CRD42022331385).

Significant outcomes

- The neurobiological underpinnings of cognitive side effects after electroconvulsive therapy are not well understood. Increased blood–brain barrier (BBB) permeability has been considered as an explanation to the cognitive side effects related to the treatment.
- Studies in animals support increased BBB permeability of some smaller molecules after modified electroconvulsive stimulation, while this is not the case in the two existing studies of humans.

Limitations

- Few studies have investigated the role of the blood–brain barrier (BBB) in the cognitive side effects associated with electroconvulsive therapy limiting the conclusions of this review.
- No studies have applied state-of-the-art methods, e.g., dynamic contrast-enhanced magnetic resonance. In related fields, studies have examined the association between cognition and BBB permeability with these methods.

## Introduction

Electroconvulsive therapy (ECT) is one of the most efficacious treatment for severe and psychotic depression (Fink and Taylor, 2007; UK ECT Review Group, 2003), yet some patients experience troublesome cognitive side effects (Semkovska and McLoughlin, 2013; Kellner *et al*., 2020). The neurobiological underpinnings of this cognitive dysfunction after ECT are largely unknown. A better understanding of the underlying mechanisms may improve the stimulation procedure resulting in fewer side effects.

There is strong evidence that a series of ECT causes grey matter volume (GMV) increases (Gbyl and Videbech, 2018a; Ousdal *et al*., 2020), most consistently documented in the hippocampus (HC) (Nordanskog *et al*., 2010; Jorgensen *et al*., 2016a; Wilkinson *et al*., 2017; Takamiya *et al*., 2018; Nuninga *et al*., 2020a; Gbyl *et al*., 2021a). The hippocampal volume has been reported to increase as early as two hours after the first ECT session (Brancati *et al*., 2021). The HC is critical for memory, including autobiographical memory, which is a major concern to patients (Rose *et al*., 2003) and to health care providers around the world in the context of ECT (Semkovska and McLoughlin, 2013; Sackeim, 2014). Several studies have linked the increase in hippocampal volume with memory impairment (van Oostrom *et al*., 2018; Argyelan *et al*., 2021; Gbyl *et al*., 2021b). The rapid volume increase in the HC could reflect a fluid shift subsequent to blood–brain barrier (BBB) alterations or neuroinflammation. There are some indications of neuroinflammation after ECT (van Buel *et al*., 2015), and the impact of ECT on the BBB has been considered in many recent ECT studies (Bouckaert *et al*., 2016; Gryglewski *et al*., 2020; Nuninga *et al*., 2020b; Repple *et al*., 2020; Brancati *et al*., 2021; Maffioletti *et al*., 2021).

In health, the BBB regulates the concentration of ions, macromolecules, nutrients, and neurotransmitters in the central nervous system (CNS) and protects the internal environment from toxins (Abbott *et al*., 2010; Sweeney *et al*., 2019). The BBB is constituted of tight junction-linked endothelial cells surrounded by pericytes, a fibroblast-like cell type, microglia, and nerve terminals (Abbott *et al*., 2010; Sweeney *et al*., 2019). The association between BBB permeability and cognition is evident in related fields. In two studies of patients with mild cognitive impairment, increased BBB permeability was associated with cognitive dysfunction (Montagne *et al*., 2015; Nation *et al*., 2019). In another study following 57 healthy subjects over 12 years, significant relations between memory retrieval and BBB leakage in both white and grey matter were found (Verheggen *et al*., 2020).

While it is known that the integrity of the BBB is reduced in areas important to memory and depression after excessive use of electroconvulsive stimulation (ECS) in animals (Bolwig, 1988; Ito *et al*., 2017), it has not been established if the BBB is affected by modern ECT. Authors often quote a narrative review from 2014 (Andrade and Bolwig, 2014) when arguing that ECT affects the BBB, but no recent or systematic review on the topic is, to our knowledge, available. On this basis, we provide a systematic and updated review of the impact of modified ECT on BBB permeability.

## Materials and methods

We present a systematic review of studies of BBB permeability after ECT adhering to the Preferred Reporting Items for Systematic Reviews and Meta-Analyses (PRISMA) statement (Page *et al*., 2021). The protocol can be accessed in the supplementary materials and was registered on PROSPERO before data extraction (CRD42022331385). Discrepancies exist between the protocol registered at PROSEPRO and the final protocol; these include a precise definition of the measurement of BBB permeability and adaption of the quality assessment to the type of studies that were included. In our opinion, these changes did not affect the conclusion of the review.

### Objective

The objective was to examine if electrically induced seizures affect the permeability of the BBB in animals (ECS) and humans (ECT).

### Litterature search

The search strategy used in PubMed/MEDLINE and Embase is presented in the supplementary materials. No filters were used. Doublets were removed automatically with Covidence software. The searches were performed on 5 April 2022 and updated 16 November 2022. The PRISMA flow chart illustrates the latter. Reference lists of included studies were scrutinised for additional papers.

### Study selection, eligibility criteria, and data collection

We included studies with a direct measurement of BBB permeability in animals (healthy or with a psychiatric disease model) treated with modified ECS and in humans (healthy or with a psychiatric disease) treated with ECT. Synthesis of results was narrative due to the low number of studies and differences in study designs. We excluded comments or letters to the editor, grey literature, conference abstracts, and non-English articles. Additional details are provided in the protocol.

We use the term BBB for both the BBB itself, the blood-cerebrospinal fluid (CSF) barrier, and the blood-spinal cord barrier in this article thus disregarding the exact pathway of transport into the CNS.

*Modified* ECS was defined as the use of anaesthesia, muscular relaxation, and ventilation mimicking ECT in humans. We defined a *direct measurement of BBB permeability* as the calculation of either the permeability, the permeability surface area-product (PS product), the transfer coefficient, K_i_, the ratio between the concentration of albumin in CSF and serum or plasma, or the presence of an injected tracer in the CNS that were not present before treatment or not present in controls. All studies with a clearly described method to measure BBB permeability not explicitly stated here were also included. A record of excluded studies is available in the supplementary materials along with the reasons for exclusion and information on whether authors were contacted.

Identified records were screened by one of the authors (CL) reading their titles or abstracts. The full texts of the chosen studies were assessed for inclusion by two authors (CL, PV) in accordance with the defined criteria. Disagreement was solved by consensus.

Data were extracted by one author (CL) and verified by a second author (PV).

### Assesment of risk of bias and methodological quality

Only clinical studies were assessed for risk of bias and methodological quality.

Pre-post studies were assessed with the National Institute of Health quality assessment tool of before-after (pre-post) studies without control group (“National Institute of Health Quality Assesment Tool for Before-After (Pre-Post) Studies With no Control Group,” 2021). Raters adhered to the tool guidelines. Evaluations were undertaken by two reviewers (CL, PV). Disagreement was solved by consensus.

## Results

A total of two human studies (31 individuals) and four animal studies (unknown total number of animals) were identified. The PRISMA flow diagram is available in the supplementary materials.

### Animal studies

Animal studies are presented in Table 1. Two studies examined the permeability of the BBB to albumin visualised with the albumin marker Evan’s blue after a single ECS (Petito *et al*., 1977; Bolwig *et al*., 1977a). One of the studies found staining of brain parenchyma of 0-1 quantified grossly from 0 to 4 (Petito *et al*., 1977), while the other found no increased permeability to albumin and no difference between controls and treated animals (Bolwig *et al*., 1977a). The latter research group used a protein tracer with a reaction product visible in electron microscope, horseradish peroxidase, in another study and found extravasation of horseradish peroxidase present in 7 out of 10 animals after a single ECS (Bolwig *et al*., 1977c). The extravasations were mainly seen around larger vessels. Control animals had no extravasations. The authors attributed the findings to increased vesicular transport caused by acute hypertension.

**Table 1.** Animal studies

Study	(Preskorn *et al*., 1981)	(Petito *et al*., 1977)	(Bolwig *et al*., 1977c)	(Bolwig *et al*., 1977a)
Number of animals				Not known
Controls	12	5		
Single ECS	12	5		
ECS series	6		10	
Type of animals	Sprague-Dawley male rats	Wistar male rats	Wistar male rats	Wistar male rats
Intervention	Single ECS or daily ECS for eight days	Single ECS	Single ECS	Single ECS
Study design	Double-labelling technique (butanol and water)	2% Evan’s blue (1.5 ml) before ECS	200 mg horseradish peroxidase before ECS	2% Evan’s blue (3 ml/kg) before ECS
Time of post-measurement (after stimulation)	15 min	20 min	3 min	15 min
ECT parameters				
Current	65 mA	100 mA	50 mA	50 mA
Voltage		100 V		
Stimulation time	1 s	1 s	0.3 s	0.3 s
Study outcome				
Increased permeability				
Single ECS	+	+/−	+	−
ECS series	+			

ECS: Electroconvulsive stimulation.

One study (Preskorn *et al*., 1981) examined the BBB permeability to water after ECS. They utilised a mathematical association (PS = − ln(1 − E_w_) x CBF, where E_w_ is the extraction of water) between the PS product and cerebral blood flow (CBF) to calculate the PS product under various CBF conditions.

The results of the study are thus mathematical associations between the PS product and CBF. At low CBF values (e.g., 50 ml per 100 g per minute), the study found the PS product of water increased after a single session as well as after an ECS series.

### Human studies

Clinical studies are presented in Table 2. One study measured changes in the CSF/serum albumin ratio before and after a series of ECT in nine patients with depression one day after the last treatment (Zachrisson *et al*., 2000). They found unchanged ratios and therefore no signs of increased BBB permeability to albumin after ECT.

**Table 2.** Human studies

Study	(Bolwig *et al*., 1977b)	(Zachrisson *et al*., 2000)
Type of study	Pre-post interventional study	Pre-post interventional study
Participants	22	9
Gender (male/female)	22/0	8/1
Age (years)	36 to 68	27 to 76
Diagnosis	Endogenous depression	MDD (7), bipolar type 2 (2)
Study design	Double indicator single injection method	CSF/serum albumin ratio
Time of post-measurement	Immediately after ECT	1 day after ECT
Medication status	Not known	Antidepressants and mood stabilisers
Intervention	Single ECT (either 4^th^ or 5^th^ treatment in a series)	ECT series of 6 treatments in 2 weeks
ECT parameters		
ECT apparatus	Siemens Konvulsator 622	Siemens Konvulsator 622
Electrode placement	Bitemporal	Unilateral
Pulse type	Not known	Unidirectional square-wave
Seizure time	30 to 55 s	39.8 s
Current	60–160 mA	0.8 A
Anaesthetics	Enibomal sodium and pancuron bromide	Methohexital, glycopyrrolate and succinylcholine
Study outcome		
Increased permeability	–	–
Study quality	Fair	Fair

ECT: Electroconvulsive therapy, MDD: Major depressive disorder, CSF: Cerebrospinal fluid.

In the other study (Bolwig *et al*., 1977b), the BBB permeability of a single passage of various radioactive smaller tracers was studied exclusively during or immediately after ECT while measuring CBF using a radioactive Xenon intraarterial injection in 22 patients. During seizure, the study found significantly increased PS product of ^14^C-urea and non-significantly increased PS products of both ^24^Na^+^, ^36^Cl^-,^ and ^14^C-thiourea. There were no dissociations of the curves between tracer and reference substances. The PS products of all tracers but thiourea were insignificantly decreased 6 min after seizure. In contrast, the PS product of thiourea was marginally increased. 15 min after the seizure, the PS product of all tracers was grossly unchanged from baseline values (the PS product of thiourea was not measured or presented). The authors conclude that the increase in transport across the BBB was not likely due to a ‘breakdown’ of the BBB but due to increased surface area of the vessels in the brain caused by changes of CBF and not the epileptic activity. The authors suggest that the mechanism behind the observed changes in PS product may be a stretching of endothelial cells in the cerebral vessels or an opening up of new capillaries, or a combination of both.

### Quality assesment

Assessment was performed with the National Institute of Health quality assessment tool of before-after (pre-post) studies without control group. Both studies (Bolwig *et al*., 1977b; Zachrisson *et al*., 2000) were assessed to be of fair quality (range: good, fair, or poor). The quality of the study by Zachrisson and co-workers is limited by a small sample size, the lack of information on whether all eligible participants that met study criteria were enrolled, and that no data on loss-to-follow are available. The study of Bolwig and co-workers is limited by a small sample size, insufficient information on eligibility criteria, and whether all eligible participants that met study criteria were enrolled.

## Discussion

We examined whether electrically induced seizures affect the permeability of the BBB in animals and humans. Three of the animal studies reported increased BBB permeability, whereas the included human studies did not find increased BBB permeability to various substances after ECT.

### Choice of methods

Our review is pragmatic; we wanted to provide an overview of studies that could be translated into clinically meaningful conclusions. To do this, we exclusively included animal studies with treatment circumstances comparable to modified human ECT, and we did not review the type of tracer transport across the BBB or the mechanisms underlying these changes. Moreover, we used the term *permeability* to describe any type of transport from the blood to the CNS disregarding if the transport was a result of a breakdown of the BBB integrity or not. Finally, we excluded studies with indirect measurements of BBB permeability. The distinction between indirect and direct measurements is arbitrary. The problem is that the cause of changes in blood and CSF concentrations of biomarkers after ECT can be difficult to elucidate. Factors that can influence biomarker concentration include changed BBB permeability, CNS vascular area, CSF-flow, or increased production or resorption of the biomarker either in the CNS or in peripheral tissue. Changes in concentrations are, therefore, not necessarily a measurement of BBB permeability. We decided only to include the ratio between the concentration of albumin in CSF and serum or plasma as a direct measurement of BBB permeability as albumin is blood derived (Reiber, 2003), and its synthesis is not expected to be influenced by ECT. Excluded studies measuring CSF total protein are mentioned later in the discussion.

### Our conclusions in relation to previous reviews

Our review is partially in line with the narrative review from 2014 (Andrade and Bolwig, 2014). Both reviews found that increased BBB permeability in animals after ECS is evident even in modified treatment conditions. The reviews differ in conclusions regarding the human studies. Andrade and co-workers conclude that some, but not all, human studies find ECT to be associated with mild transient brain oedema and penetration of some substances to the CSF. The reason for this discrepancy between the reviews is that Andrade and co-workers included studies with indirect measurements of BBB permeability and that we distinguished between the terms permeability and PS product. The differences are further elaborated in the following.

### Animal models of ECT

Three out of four animal studies found that, even in modified conditions, increased BBB permeability to various tracers is present after ECS. The studies have some methodological limitations; Evan’s blue may bind to other proteins than albumin or remain unbound, and horseradish peroxidase may cause degranulation of mast cells affecting vascular permeability (Saunders *et al*., 2015).

The studies that used Evan’s blue found either marginally increased or unchanged permeability to albumin. Contrary to this, a single ECT increased the permeability of the BBB to horseradish peroxidase and water (at lower CBF values). Albumin is a much larger molecule than horseradish peroxidase and water. These results thus suggest that a single ECS increases the BBB permeability of some smaller molecules.

### Human studies

The human studies included in this systematic review found no changes in the permeability of the BBB to various substances. Although Bolwig and co-workers found significantly increased PS product of ^14^C-urea during ECT, it is important to notice that the study found similar changes to the PS product during an artificial increase of CBF to ECT levels by increased arterial CO_2_. Although permeablity and vascular surface area cannot be clearly separated in the PS product, the results suggest that the increased CBF during seizure increases the surface area of the brain capillaries with a subsequent increase of substance transport across the BBB. However, the conclusion that the BBB permeability is unchanged after ECT cannot be made for several reasons. First, both studies may be underpowered. Second, in the study by Bolwig and co-workers, as pointed out by the authors, the measurement was performed after a single passage of the brain vasculature by the test substances. A marginally increased permeability would therefore be more evident with increased number of passages. Finally, Zacharrison and co-workers measured the CSF/serum albumin ratio the day after ECT; if the change in BBB permeability is temporary, it may be too late to detect any changes.

### Biomarker studies as indication of BBB permeability

We excluded a case report included in the narrative review of Andrade and co-workers that found the concentration of CSF total protein increased three days after ECT in a physically healthy patient (Alexopoulos *et al*., 1978). The CSF protein concentrations were normalised within two weeks.

The narrative review quotes one study measuring amyloid beta peptides and one measuring brain-type creatine phosphokinase (Andrade and Bolwig, 2014). Three studies found no changes in serum brain-type creatine phosphokinase after ECT (measured from 5 min to 3 days after ECT) (Taylor *et al*., 1981; Webb *et al*., 1984; Giltay *et al*., 2008). One study (Zimmermann *et al*., 2012) found a significant increase in amyloid beta concentration 0.5 h after ECT, which were normalised 2 h later, while another study (Piccinni *et al*., 2013) found no change in concentration one week after ECT.

The glial cell protein, S100B, has been shown to provide reasonable estimates of BBB permeability in other fields (Kapural *et al*., 2002; Kanner *et al*., 2003). However, the use of this protein as a biomarker of BBB alterations after ECT in psychiatric diseases has some limitations. First, some evidence suggests that its serum level may be influenced by the depressive state (Kroksmark and Vinberg, 2018; Tural *et al*., 2022). Second, psychotropic medication may also have an impact (Schroeter *et al*., 2013; Kroksmark and Vinberg, 2018). Third, various CNS disorders are associated with a rise in serum S100B, including stroke, neurodegenerative diseases, and traumatic brain injury (Dassan *et al*., 2009; Ercole *et al*., 2016; Michetti *et al*., 2019). Finally, the half lifetime of this molecule is only 1 to 2 hours (Ingebrigtsen and Romner, 2002), which reduces the chance of detecting a possible increase after ECT. Several studies investigated the effect of ECT on serum S100B (Agelink *et al*., 2001; Palmio *et al*., 2010; Kranaster *et al*., 2014; Gbyl *et al*., 2022; Arts *et al*., 2006). Only one of them (Arts *et al*., 2006) found a small and temporary increase in serum S100B 1h after a single ECT session. Thus, most of these ECT studies did not find evidence for an increase in serum S100B, although many of them measured the concentration acutely, i.e., hours after a single ECT session.

### Magnetic resonance imaging (MRI) studies of BBB permeability

In vasogenic oedema, water accumulation in the extracellular space facilitates an unrestricted movement of water molecules, leading to increased mean diffusivity (MD). This parameter can be measured using diffusion tensor imaging (DTI). All three studies investigating ECT-related MD changes in the HC, however, found a significant MD decrease (Jorgensen *et al*., 2016b; Yrondi *et al*., 2019; Nuninga *et al*., 2020b). Furthermore, MD decreases were also observed in the hypothalamus (Jorgensen *et al*., 2016b) and the amygdala (Yrondi *et al*., 2019).

All three mentioned DTI studies measured MD within one week after an ECT series. Thus, they were not designed to detect oedema that might have occurred acutely, i.e., within hours after a single ECT session. Fortunately, another type of studies measuring relaxation times investigated the acute effects. They took advantage of the fact that elevated brain water content increases T1 and T2 relaxation time. As presented in our previous review (Gbyl and Videbech, 2018b), two relaxometry studies did not find any indications of oedema, which contrasted with three other studies detecting a transient increase in T1 relaxation time within six hours after a single ECT session. However, the latter studies had several methodological limitations. Two of these studies are quoted in the narrative review by Andrade and co-workers (Andrade and Bolwig, 2014). Furthermore, as assessed by visual inspection, no clear sign of hippocampal oedema was found within five hours after a single ECT session in a study using diffusion-weighted imaging (DWI). This MR modality is highly sensitive to detecting oedema-related signal abnormalities (Szabo *et al*., 2007), but more advanced techniques may be required to visualise vasogenic oedema.

In addition to these MRI studies, we excluded the only study that used a Gadolinium-based MRI contrast agent because the use of modified ECT was not described. The case report found Gadolinium-based contrast extravasations in the subarachnoid space after a single ECT in the brain of a 70-year-old man suffering from treatment-resistant depression (Taydas *et al*., 2018). No information of co-morbid diseases is presented in the case report. The contrast agent was injected on the day of ECT as part of a routine MRI scan to exclude organic course of the depression. The day after ECT, the patient developed headache suggesting subarachnoid haemorrhage. As part of the evaluation, an MRI without contrast was carried out. No cause of the headache was found, and the cause of the contrast extravasations was concluded to be ECT.

## Strengths and limitations

Our review is, to our knowledge, the first systematic review with quality assessments of BBB permeability after ECT. We performed a pragmatic review prioritising research approaches that could be translated into clinically useful conclusions; we included studies with a direct measurement of BBB permeability and excluded animal studies with unmodified or excessive ECS. This strength comes with the disadvantage of a limited number of studies.

Our conclusions are limited by the low number of studies and the low number of subjects and animals. Despite our use of restrictive criteria, the included studies have methodological differences in the methods applied to the measurement of BBB permeability, the time of measurement, and the subjects included making synthesis of the results challenging.

## Conclusion

In animals, studies support increased BBB permeability to smaller molecules after modified ECS. Only two human studies with a direct measurement of BBB in humans after modified ECT have been identified. They do not support increased BBB permeability to urea, Na^+^, Cl^−^, or thiourea during or after ECT or to albumin after ECT. More studies with modern approaches to examination of BBB permeability are warranted.

## Future directions

Although our review does not provide support of an increased BBB permeability after ECT, the limitations confine the conclusions. As described, the association between BBB permeability and cognition is evident in related fields. In combination with the results of the animal studies in this review, this calls for future studies of BBB and cognition in relation to ECT using modern methods, e.g., dynamic contrast-enhanced MRI.

## Supplementary material

The supplementary material for this article can be found at https://doi.org/10.1017/neu.2023.48.
